# Efficacy of intravenous immunoglobulin in recurrent pregnancy loss: a retrospective analysis of patients with abnormal cellular immunity

**DOI:** 10.3389/fendo.2025.1546602

**Published:** 2025-06-04

**Authors:** Jae Won Han, Jin Sol Park, Jong-Seok Kim, Sung Ki Lee

**Affiliations:** ^1^ Department of Obstetrics and Gynecology, Konyang University College of Medicine, Daejeon, Republic of Korea; ^2^ Myunggok Medical Research Institute, Konyang University College of Medicine, Daejeon, Republic of Korea

**Keywords:** recurrent pregnancy loss, immunity, natural killer cells, natural killer cell cytotoxicity, TH1/TH2 ratio, intravenous immunoglobulin

## Abstract

**Introduction:**

Various causes of recurrent pregnancy loss (RPL) have been identified, but even with a detailed evaluation, almost half of the cases have unidentified etiologies. Immune imbalance is one of the proposed potential etiologies of these idiopathic RPL. To regulate abnormal cellular immunity, intravenous immunoglobulin (IVIG), a type of immunotherapy, is proposed to improve pregnancy outcomes. However, the efficacy of IVIG in RPL is still controversial.

**Methods:**

RPL was defined as women with two or more spontaneous abortions and in total, 987 RPL women visited Department of Obstetrics and Gynecology, Konyang University Hospital from January 2007 to December 2020. Only those with a full evaluation and known treatment outcome were included. Idiopathic RPL(n=215) and women with known etiology (n=251) were enrolled. Both the idiopathic and known etiology groups were subsequently stratified into subgroups based on the presence of at least one abnormal cellular immunity (n=100 and n=97, respectively). We investigated the pregnancy outcome by sorting the patients into seven subgroups depending on abnormal cellular immunity including natural killer (NK) cell level, NK cell cytotoxicity and Th1/Th2 ratio.

**Results:**

Patients with older age and higher body mass index had negative effect on pregnancy outcomes whereas the number of previous miscarriages did not show significant difference in pregnancy outcomes. Among all RPL women with at least one abnormal cellular immunity were treated with IVIG and the overall live birth rate (LBR) was 82.7%. The group which did not have IVIG treatment showed an overall LBR of 80.7%. Among the seven groups of idiopathic RPL women with abnormal cellular immunity, the group with both high NK cell level and NK cell cytotoxicity showed the highest LBR, 90.5%, and the group with both high NK cell level and Th1/Th2 ratio showed the lowest LBR, 75%.

**Discussion:**

IVIG treatment appears to improve LBRs in women with RPL and abnormal cellular immunity. These findings support the potential benefit of IVIG in selected RPL patients with immune imbalances. Further studies are needed to refine patient selection criteria and optimize treatment protocols for improving pregnancy outcomes in this population.

## 1 Introduction

Spontaneous abortion can be a distressing experience for individuals attempting to conceive, with approximately 15 to 30% of pregnancies ending in miscarriage before reaching 20 weeks of gestation (1, 2). Recurrent pregnancy loss (RPL), though widely studied, remains variably defined across international guidelines. The American Society for Reproductive Medicine (ASRM) defines RPL as two or more failed clinical pregnancies documented by ultrasound or histopathologic examination (3), whereas the European Society of Human Reproduction and Embryology (ESHRE) sets the threshold at three or more consecutive pregnancy losses (4). This lack of uniformity poses challenges in patient counseling, research standardization, and treatment recommendations. As such, definitions of RPL remain a subject of ongoing debate and the prevalence are approximately 1% with three consecutive abortions and up to 5% with two consecutive abortions (5, 6). Various factors contributing to RPL have been identified, such as anatomical anomalies, endocrine disorders, genetic abnormalities, infections, and autoimmune issues. Nonetheless, even after thorough evaluations, nearly half of RPL cases have unknown causes (7, 8). Among the multifactorial causes of RPL, immune imbalance has emerged as a plausible but controversial etiology, especially in idiopathic RPL (7–9). Pioneering studies by Kwak-Kim et al. and others have highlighted aberrant immune responses such as elevated natural killer (NK) cell proportions or cytotoxicity, increased Th1/Th2 or Th17/Treg ratios as potential contributors to early pregnancy loss (10–14). Recently, international societies have begun to reflect a more positive perspective on immune testing in recurrent pregnancy loss (RPL) by publishing guidelines and reviews on this subject (4, 15). Moreover, an international web-based survey of IVF clinicians reported that 69% of respondents recommend immunologic testing for patients with RPL (14). This finding suggests that, despite ongoing debates, clinical practice appears to reflect growing interest in immune testing. The immune system’s delicate balance is therefore essential in promoting successful pregnancy.

Mor et al. outlined the maternal immune adaptation across pregnancy as three immunological stages (11): the first trimester characterized by a pro-inflammatory state, the second trimester marked by an anti-inflammatory phase, and finally, a return to a pro-inflammatory state triggering labor and delivery. The immune system undergoes intricate interactions during pregnancy to support a healthy and successful gestation, and effective immune regulation during early pregnancy is a critical factor for successful gestation. Although many clinicians acknowledge the potential role of immune factors in early pregnancy, there is no universal consensus regarding their diagnostic markers or therapeutic targets. Consequently, standard diagnostic tools and treatment strategies regarding immune imbalance in RPL women remains elusive.

Several studies have indicated that women with RPL often show a higher proportion and cytotoxicity of natural killer (NK) cells in peripheral blood which can serve as a valuable predictor of poor pregnancy outcomes (12, 16–23). Yamada et al. also found that elevated NK cell levels before pregnancy could predict spontaneous abortion (24). In cases of spontaneous abortion with normal fetal karyotype or biochemical pregnancies, RPL women exhibited significantly higher NK cell percentages compared to those with successful live births. Additionally, an increased ratio of Th1/Th2 and Th17/Treg has been implicated as a contributing factor to adverse pregnancy outcomes (13, 25–27). We identified three cellular immune markers to assess immune abnormalities—NK cell proportion, NK cell activity, and Th1/Th2 ratio—before pregnancy in women with RPL and determined their cutoff values (23).

As immunotherapy, IVIG, has been used to treat various autoimmune and inflammatory diseases, IVIG has also been considered as a potential treatment for RPL patients with immune abnormalities (2, 20, 28–36). Numerous studies have demonstrated that IVIG treatment improved pregnancy outcomes by regulating elevated levels of NK cells, cytotoxicity, and Th1/Th2 ratios (29, 32, 36, 37). Moraru et al. reported a beneficial effect of IVIG therapy in patients with recurrent miscarriage or recurrent implantation failure (RIF) who showed elevated NK cell levels (defined as >12%). Forty women received IVIG treatment, while a control group of 24 women did not receive IVIG. As a result, the LBR was significantly higher in the IVIG group compared to the control group (82.5% vs. 12.5%; P < 0.0001; OR = 34) (28). The latest ESHRE guideline has revised the use of IVIG in women with RPL, adopting a more favorable stance (2, 38). While its recommendation remains restricted to women with four or more miscarriages, the shift reflects a growing recognition of IVIG as a potential therapeutic option. However, the use of IVIG still remains controversial, primarily due to inconsistent findings and heterogeneity across studies due to a lack of standardized patient selection criteria and different cutoff values of these markers (37, 39–43).

This study aims to evaluate the efficacy of IVIG treatment in women with RPL and abnormal cellular immunity, with a particular focus on individual immune markers—namely, NK cell level, NK cell cytotoxicity, and the Th1/Th2 ratio—as well as combinations of these parameters. Given that previous studies have reported only fragmented and limited findings regarding outcomes by immune profile, we sought to conduct a more detailed analysis through patient stratification. Our goal was to determine whether the effectiveness of IVIG varies depending on specific immune abnormalities and, in doing so, to clarify its role in improving LBRs and identify subgroups of patients who are most likely to benefit from this therapeutic approach.

## 2 Materials and methods

### 2.1 Patient enrollment and evaluation

**Figure 1:**
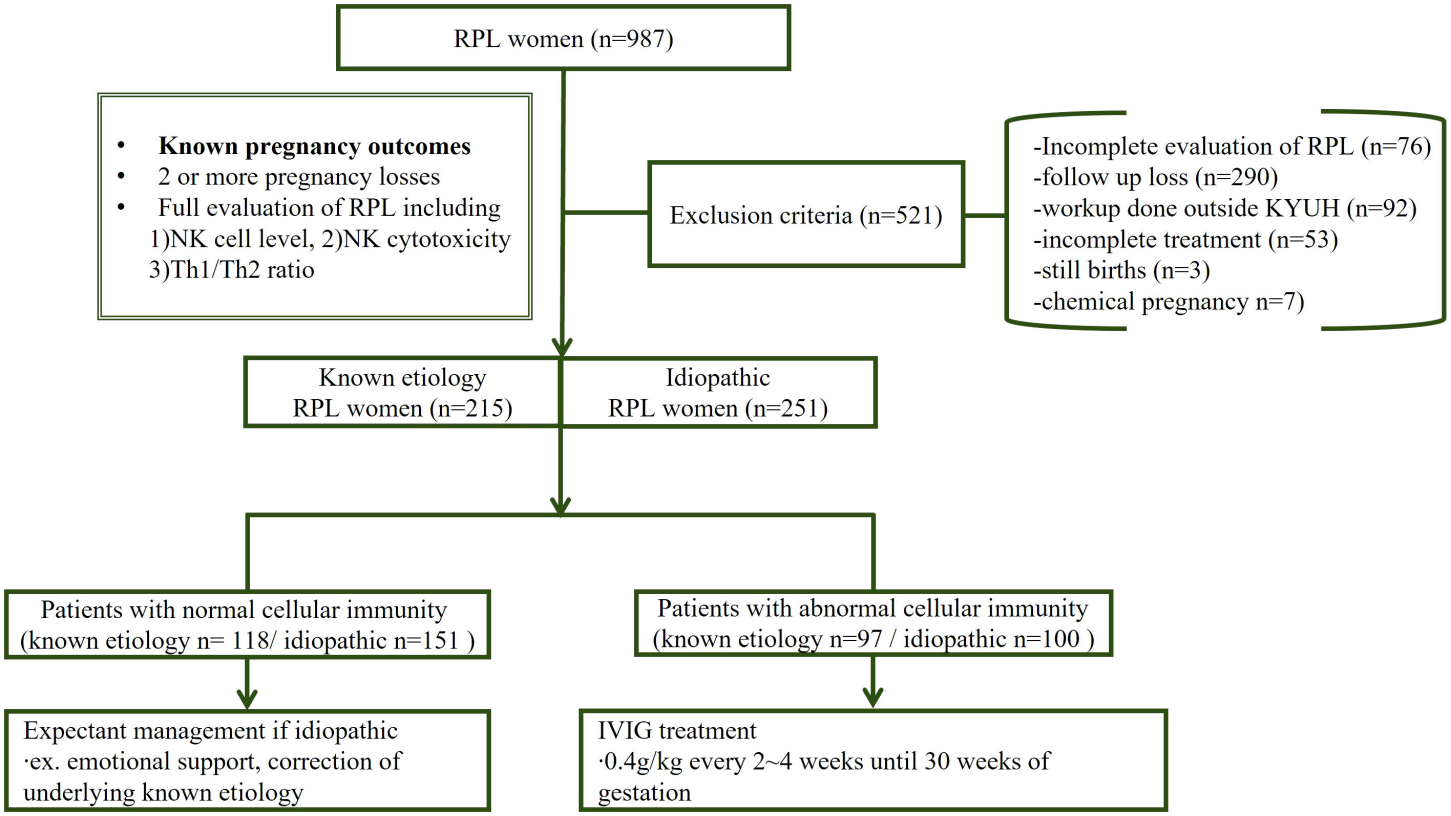
Patient enrollment. Among the RPL patients who visited our clinic, those meeting the exclusion criteria were excluded, and only patients with documented pregnancy outcomes were included. All RPL patients underwent a full evaluation of cellular immunity tests, and the outcome of the first pregnancy following this assessment was analyzed. The patients were then categorized into two groups: those with a known etiology and idiopathic RPL. Each group was further subdivided based on abnormal cellular immunity, patients with abnormal immunity received IVIG treatment.

The study was designed as a retrospective cohort study at Department of Obstetrics and Gynecology, Konyang University Hospital (KYUH), Daejeon. We collected the medical records of women with 2 or more spontaneous abortions as ESHRE defined (38), and total 987 women who visited our clinic from January 2007 to December 2020 were initially enrolled (**Figure 1**). We only included patients with confirmed pregnancy outcomes and with a full evaluation of the cause of RPL. Full evaluations were done before the index pregnancy. It includes parental karyotyping, image workups to exclude anatomical causes (pelvic sonography, hysterosalpingography, hysteroscopy), thyroid stimulating hormone(TSH), serum level of prolactin, free androgen index, bacterial culture from the vagina (Mycoplasma, Ureaplasma, Chlamydia) or bacterial vaginosis was diagnosed using a multiplex polymerase chain reaction (PCR) assay (Anyplex™ II STI-12 Detection kit, Seegene Inc., Seoul, South Korea), antiphospholipid antibodies (IgG and IgM anticardiolipin, lupus anticoagulant, and anti-b2 glycoprotein 1 antibodies), and antithyroid antibodies (thyroglobulin and thyroperoxida se antibodies). Peripheral blood test including cellular immune tests was taken in the early to mid-follicular phase of the menstrual cycle. Cellular immune tests included NK cell level, activity and Th1 and Th2 cytokine-producing T helper cell ratio as previously reported (23). The cutoff values for each marker were as follows: NK cell level, >16.1% of lymphocytes; high NK cell cytotoxicity at the effector-to-target cell ratio of 50:1, 25:1 and 12.5:1, >34.3%, 23.8%, and 9.6% respectively; TNF-α producing CD4^+^ T cell to IL-10 producing CD4^+^ T cell ratio, >36.2% (23).

After the exclusion, a final 466 women were included. Only the first pregnancy outcome following the baseline workup was enrolled to avoid overlapping second or third pregnancy outcomes of the same patient. Patients were divided into two groups: those with known etiologies (n=215) and idiopathic RPL (n=251). Known etiology was defined as patients with an identifiable cause of RPL based on full evaluation, including parental chromosomal abnormalities, anatomical defects such as uterine septum, endocrine disorders (e.g., thyroid dysfunction, hyperprolactinemia), infections, or thrombophilic conditions such as antiphospholipid syndrome. Both the known etiology and idiopathic groups were further categorized based on their immune profiles into those with normal or abnormal cellular immunity. Pregnancy outcome was considered successful if women delivered a live born infant in index pregnancy.

In cases with abortion outcomes, when the patient gave consent, fetal karyotyping was done to determine the cause of miscarriage.

This study received the local Institutional Review Board approval (KYUH 2020-05-017).

### 2.2 Excluded cases

Patients who had incomplete workups (n=76), workup done outside KYUH (n=92), or all follow-up losses (n=290) were excluded. Stillbirths (n=3) and chemical pregnancies(n=7) were also excluded (**Figure 1**). We considered that including stillbirths was not adequate in interpreting the results because these three cases may be related to late pregnancy events (20 weeks, 22 weeks, and 34 weeks of gestation) and are not compatible with the definition of spontaneous abortion. In cases of chemical pregnancies, although we recommend the patients visit our clinic as soon as possible to start treatment, for various reasons, patients come after that critical period. Therefore, we decided to exclude these untreated cases in assessing treatment outcomes. Also, those who did not follow our treatment protocols for personal reasons, such as the inconvenience of hospital visits and financial constraints, were excluded (n=53).

### 2.3 The treatment protocols

In the group with known etiology, identifiable causes of RPL were addressed with appropriate management prior to and during pregnancy. Anatomical abnormalities were surgically corrected before conception, confirmed infections were treated with antibiotics, and elevated prolactin levels were managed using dopamine agonists. Conditions related to abnormal thyroid function such as positive antithyroid antibodies or elevated thyroid-stimulating hormone (TSH), and thrombophilia were treated before and throughout pregnancy. For example, TSH levels were maintained below 2.5 mU/L, and patients with acquired thrombophilia—including antiphospholipid syndrome (APS)—received anticoagulant therapy with low-dose aspirin (100 mg orally, daily) and low-molecular-weight heparin (LMWH), dalteparin 2500 IU or enoxaparin 40mg, daily (32, 44). Moreover, IVIG was administered concurrently if cellular immune abnormalities were also present. Conservative care was given for couples with parental genetic factor. If abnormal cellular immunity was present, IVIG treatment was administered regardless of whether women had known etiology or were in an idiopathic group. The IVIG treatment protocol consisted of administering 0.4 g/kg of body weight immediately after pregnancy confirmation, defined by either a rise in serum β-hCG or the presence of a gestational sac on transvaginal ultrasonography (45). In patients undergoing IVF cycles, IVIG was administered on the day of oocyte retrieval or embryo transfer. Following initiation, IVIG treatment continued every 3 to 4 weeks until 30 weeks of gestation. In the idiopathic group, women with normal cellular immunity received expectant management such as emotional support.

### 2.4 Statistical analysis

Statistical analysis was performed using IBM SPSS Statistics 27. The data was presented as mean ± standard deviation and the patient characteristics were analyzed by one-way ANOVA. We applied the Scheffé method as a *post-hoc* analysis. Treatment outcomes and aneuploidy rate were analyzed with a chi-square test. P-values less than 0.05 were considered statistically significant. Moreover, logistic regression analysis was done to identify factors that influence pregnancy outcomes.

## 3 Results

### 3.1 Demographic characteristics of the study group

A total of 466 women with RPL were included in the study, categorized into two groups based on the identified cause. (**Table 1**) Both known etiology and idiopathic groups were further divided into those with normal cellular immunity and those with abnormal cellular immunity. Among the 215 women in the known etiology group, 97 had abnormal cellular immunity, while out of the 251 women in the idiopathic etiology group, 100 had abnormal cellular immunity. The mean age of participants was 32.78 ± 3.72 years in the known etiology group and 33.66 ± 3.85 years in the idiopathic group. The mean body mass index (BMI) was 23.00 ± 4.2 kg/m² in the known etiology group and 21.9 ± 3.01 kg/m² in the idiopathic group. No significant differences were observed in the number of previous miscarriages or parity across the groups. ANOVA revealed significant differences in age (F = 2.872, p = 0.036) and BMI (F = 2.7, p = 0.045) among the groups, although subsequent Scheffé’s *post-hoc* tests did not reveal any significant pairwise differences between the groups. The number of miscarriages and parity did not show significant differences between the groups. Additionally, as IVIG was only administered to the abnormal cellular immunity group, the difference was significant.

**Table 1:** Demographic features of the selected patients.

Characteristics	Known etiology (n=215)		Unknown etiology (n=251)		F value	P- value
	Normal cellular immunity (n=118)	Abnormal cellular immunity (n=97)	Normal cellular immunity (n=151)	Abnormal cellular immunity (n=100)		
Age	32.78 ± 3.72 (22-43)	32.39 ± 3.78 (25-45)	33.66 ± 3.85 (25-42)	33.42 ± 3.46 (26-42)	2.872	N-S
BMI	23.00 ± 4.2 (14.5-39.3)	21.71 ± 3.15 (16-36.1)	21.9 ± 3.01 (16.7-33.6)	21.99 ± 3.45 (16.7-33.2)	2.7	
Previous number of miscarriages	2.89 ± 1.23 (2-10)	3.18 ± 1.23 (2-8)	2.87 ± 1.13 (2-10)	2.95 ± 1.14 (2-7)	1.705	
Parity (n)	0.09 ± 0.32 (0-2)	0.11 ± 0.32 (0-1)	0.18 ± 0.42 (0-2)	0.15 ± 0.35 (0-1)	1.687	
Total IVIG dose (g)	0	114.53 ± 105.32 (20-498)	0	154.06 ± 79.18 (0-422)	189.127	0.001

BMI, body mass index; IVIG, intravenous immunoglobulin; N-S, not significant (Scheffé *post hoc* test)t.

### 3.2 Prevalence of abnormal cellular immunity

**Figure 2:**
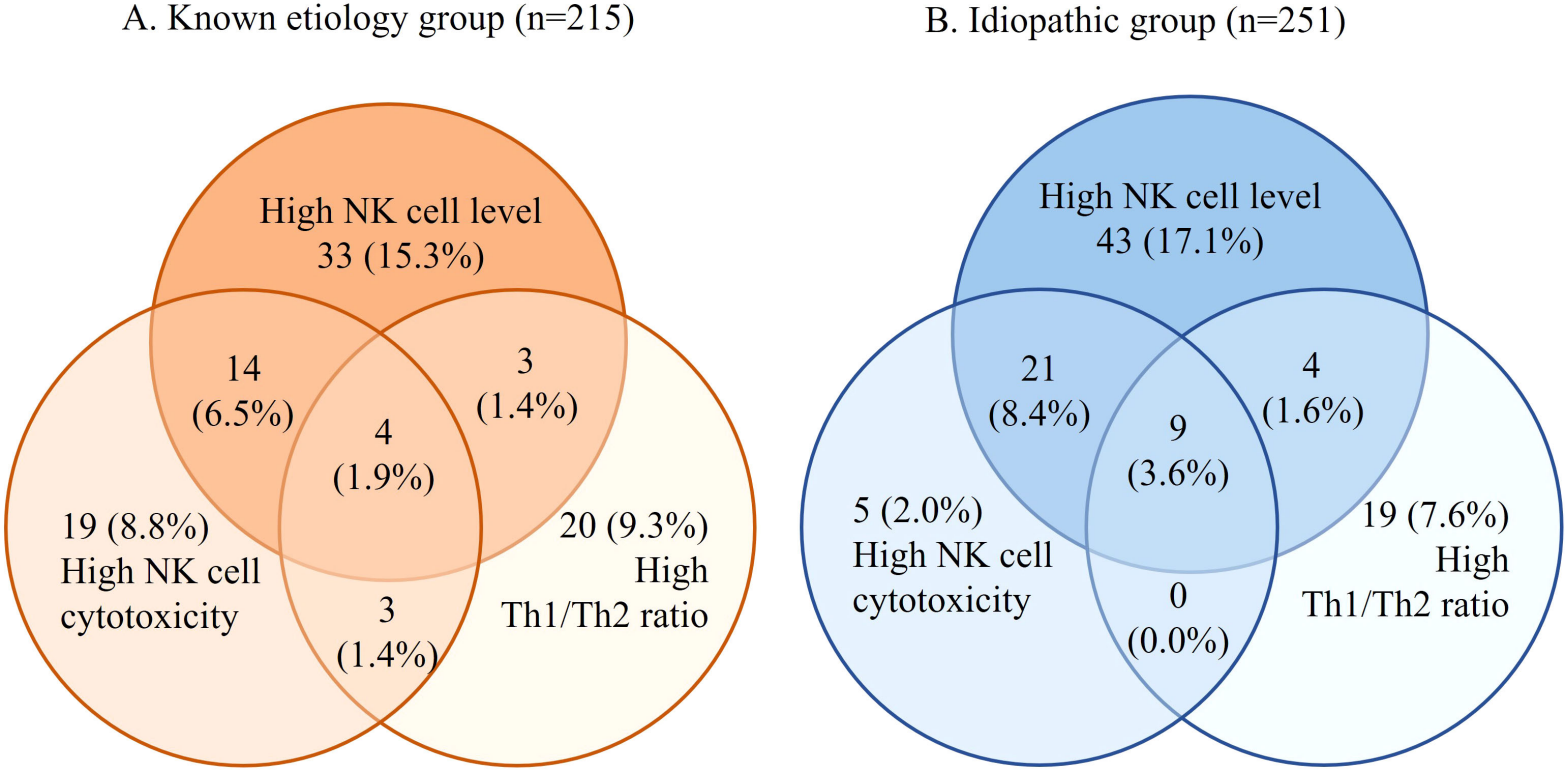
The prevalence of abnormal cellular immunity in each group of enrolled patients. **(A)** The known etiology group. **(B)** The idiopathic group. Determination of cellular immunity was based on the cutoff values that were previously reported; NK cell level, >16.1% of lymphocytes; high NK cell cytotoxicity at the effector-to-target cell ratio of 50:1, 25:1 and 12.5:1, >34.3%, 23.8%, and 9.6% respectively; TNF-α producing CD4^+^ T cell to IL-10 producing CD4^+^ T cell ratio, >36.2%.

The prevalence of abnormal cellular immunity was similar in both groups (**Figure 2**). In the known etiology group, 45.1% (n=97) exhibited abnormal cellular immunity, while in the idiopathic group, 39.8% (n=100) showed abnormalities. The difference was not statistically significant. (p=0.250) The most common immune abnormality in both groups was a high percentage of NK cells (15.3% in known etiology, 17.1% in idiopathic), followed by elevated NK cell cytotoxicity and an abnormal Th1/Th2 ratio (**Figure 2**).

### 3.3 Pregnancy outcomes

**Figure 3:**
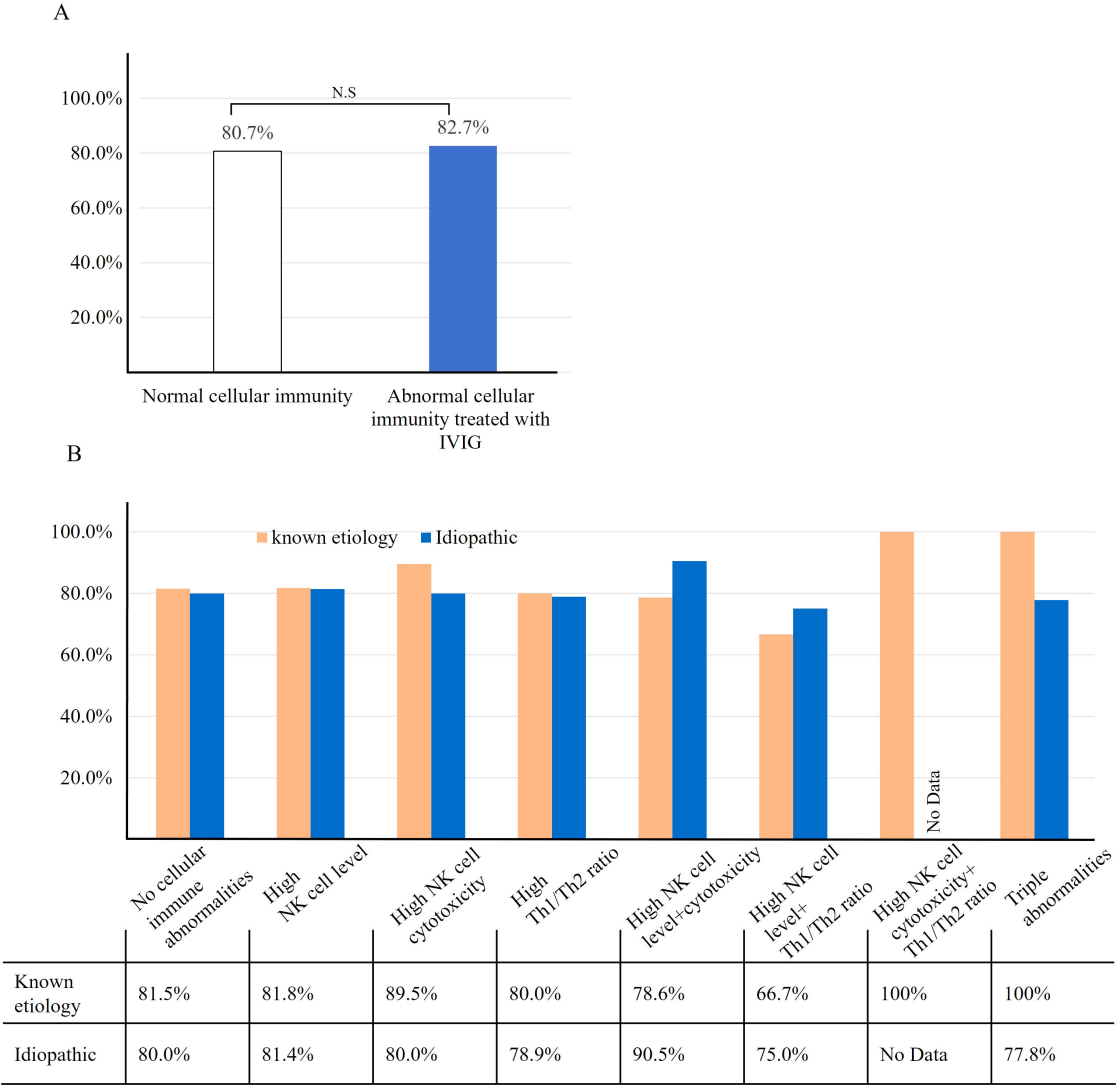
Pregnancy outcomes following etiology-based treatment. **(A)** The comparison of live birth rate between normal cellular immunity group and IVIG treated group. The live birth rate between two groups were not significant. **(B)** Live birth rate according to the types of abnormal cellular immunity. We compared the LBR between the known etiology and idiopathic groups by further subdividing the patients based on the diagnosed type of abnormal cellular immunity. The LBR among patients without abnormal cellular immunity was not significantly different between the known etiology and idiopathic groups. Additionally, no statistically significant differences in LBR were observed among the different types of abnormal cellular immunity. However, no patients presented with both elevated NK cell cytotoxicity and an abnormal Th1/Th2 ratio, precluding analysis of this specific combination. N-S, Not significant.

The overall LBR for women treated with IVIG due to abnormal cellular immunity was 82.7% (163/197), compared to 80.7% (217/269) in the normal immunity group who did not receive IVIG (**Figure 3A**). No statistically significant difference was observed in LBRs between the two groups (p =0.569).

We further analyzed the LBRs according to each marker abnormality and combinations of the markers. Among the subgroups of women with idiopathic RPL and abnormal cellular immunity, those with both high NK cell levels and NK cell cytotoxicity had the highest LBR at 90.5%, while those with high NK cell levels and an elevated Th1/Th2 ratio had the lowest LBR at 75%. None of the idiopathic RPL group had both high NK cell cytotoxicity and high Th1/Th2 ratio. The difference among the subgroups were not statistically significant (p=0.958) (**Figure 3B**) In the known etiology group, the LBRs of the subgroups were not significantly different compared to those in the idiopathic group. (p=0.931).

### 3.4 Factors affecting pregnancy outcomes

Further analysis was performed to find out the factors influencing LBRs. As a result, advanced maternal age and higher BMI were negatively correlated with LBRs (OR = 0.924, 95% CI: 0.867–0.984, p = 0.014 for age; OR = 0.920, 95% CI: 0.864–0.981, p = 0.010 for BMI). However, the number of previous miscarriages did not significantly affect LBR (p = 0.891) (**Table 2**).

**Table 2:** Factors that affect live birth rate.

	B	OR	95% CI	P-value
All RPL group				
Age	-0.080	0.924	0.867-0.984	0.014*
BMI	-0.083	0.920	0.864-0.981	0.010*
Previous number of miscarriages	0.015	1.015	0.822-1.253	0.891
Known etiology group				
Age	-0.016	0.984	0.895-1.083	0.744
BMI	0.081	0.922	0.844-1.008	0.074
Previous number of miscarriages	0.056	0.945	0.713-1.252	0.694
Idiopathic group				
Age	-0.141	0.868	0.792-0.952	0.003**
BMI	-0.093	0.911	0.828-1.003	0.056
Previous number of miscarriages	-0.131	1.140	0.816-1.593	0.441

RPL, Recurrent pregnancy loss; BMI, body mass index; B, regression coefficient; OR, Odds ratio; CI, confidence interval. (*P<0.05 **P<0.01)

### 3.5 Live birth rate according to number of previous miscarriages

**Figure 4:**
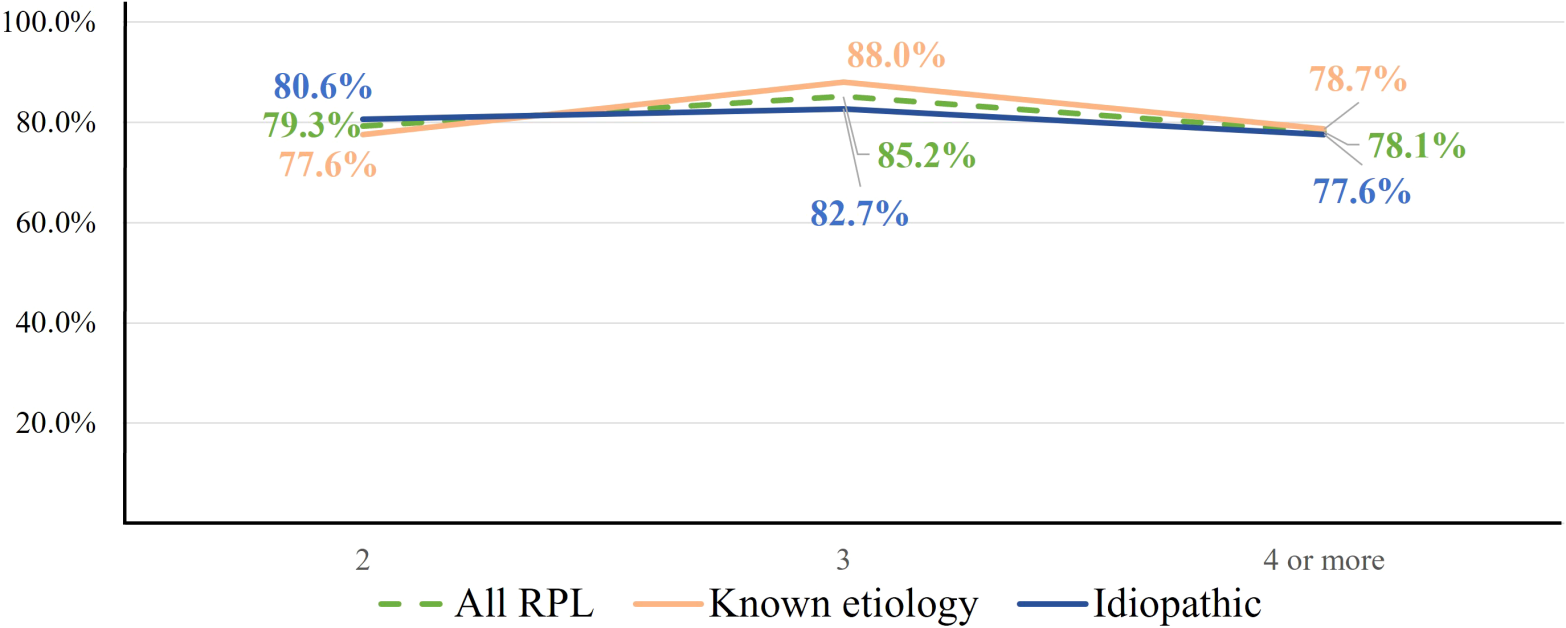
The live birth rate according to number of previous miscarriages. The LBR among all RPL patients showed a plateau up to the group with four or more miscarriages, and a similar pattern was observed in both the known etiology and idiopathic groups.

**Figure 4** displays the LBRs based on the number of previous miscarriages. Although the LBRs appeared to decrease with an increasing number of previous miscarriages, this trend did not reach statistical significance for any of the groups: all RPL (p=0.162), known etiology (p=0.750), and idiopathic RPL group (p=0.216).

### 3.6 Prevalence of RPL in women with abnormal cellular immunity according to previous number of miscarriages

We enumerated the possibility of an association between the cellular immune abnormality and the number of miscarriages because a report from Yamada et al. suggested the beneficial effect of IVIG was prominent in idiopathic RPL women with a history of four or more miscarriages (2).

**Figure 5:**
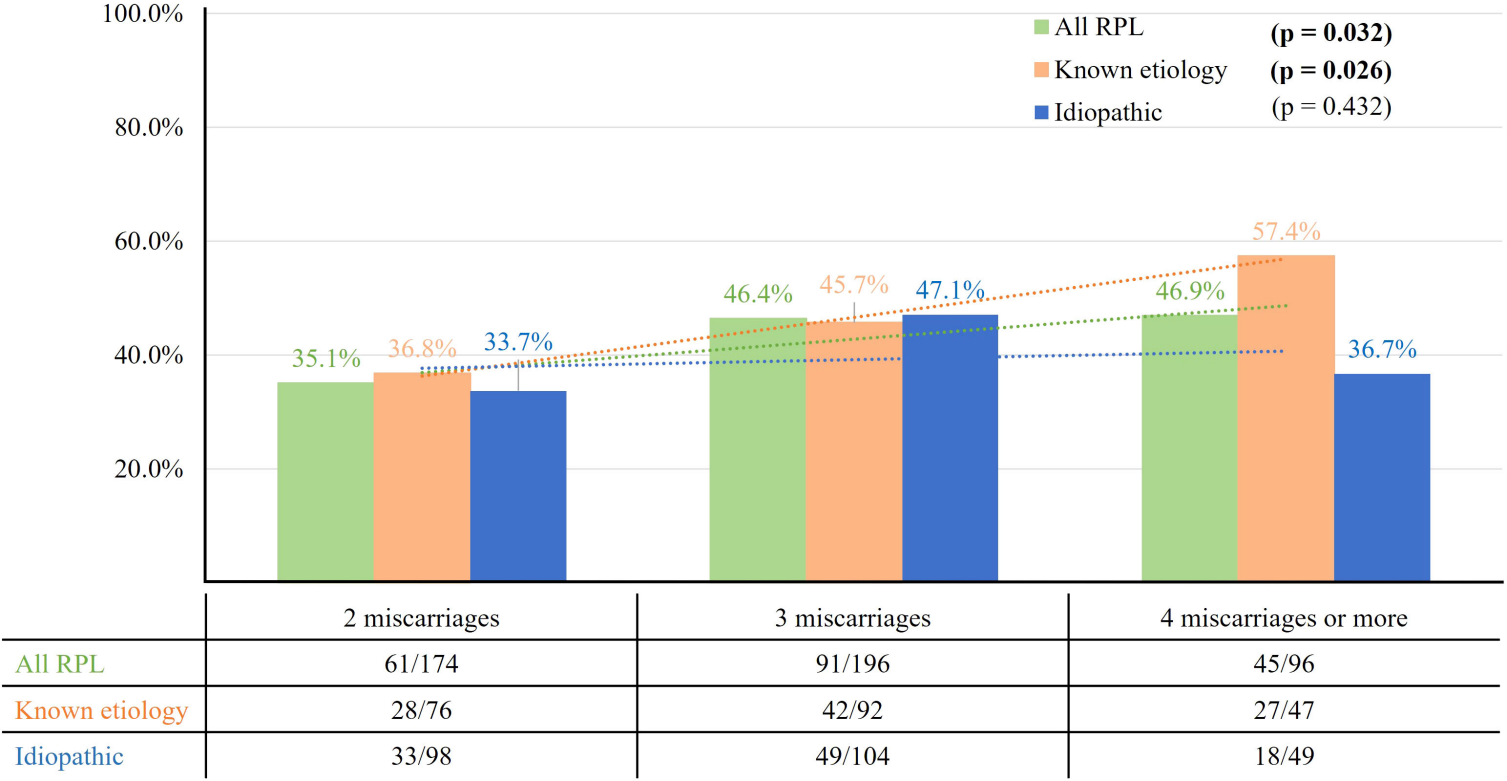
The prevalence of RPL women with at least one abnormal cellular immunity according to previous number of miscarriages. Both the all RPL group and the known etiology group demonstrated a significant increasing trend (p = 0.032 and 0.026, respectively). In contrast, no significant trend was observed in the idiopathic group.

Examining the relationship between the prevalence of abnormal cellular immunity and the number of previous miscarriages suggested a potential increase in immune abnormality with a higher number of previous miscarriages in all RPL women. (**Figure 5**) All RPL group and known etiology group both showed a trend, a significant increase of cellular abnormality as the number of miscarriages increased. (All RPL p=0.032, known etiology p=0.026) However, the idiopathic group did not show any trend. (p=0.432).

### 3.7 Correlation between each type of cellular immunity and number of previous miscarriages in known etiology and idiopathic groups

**Figure 6:**
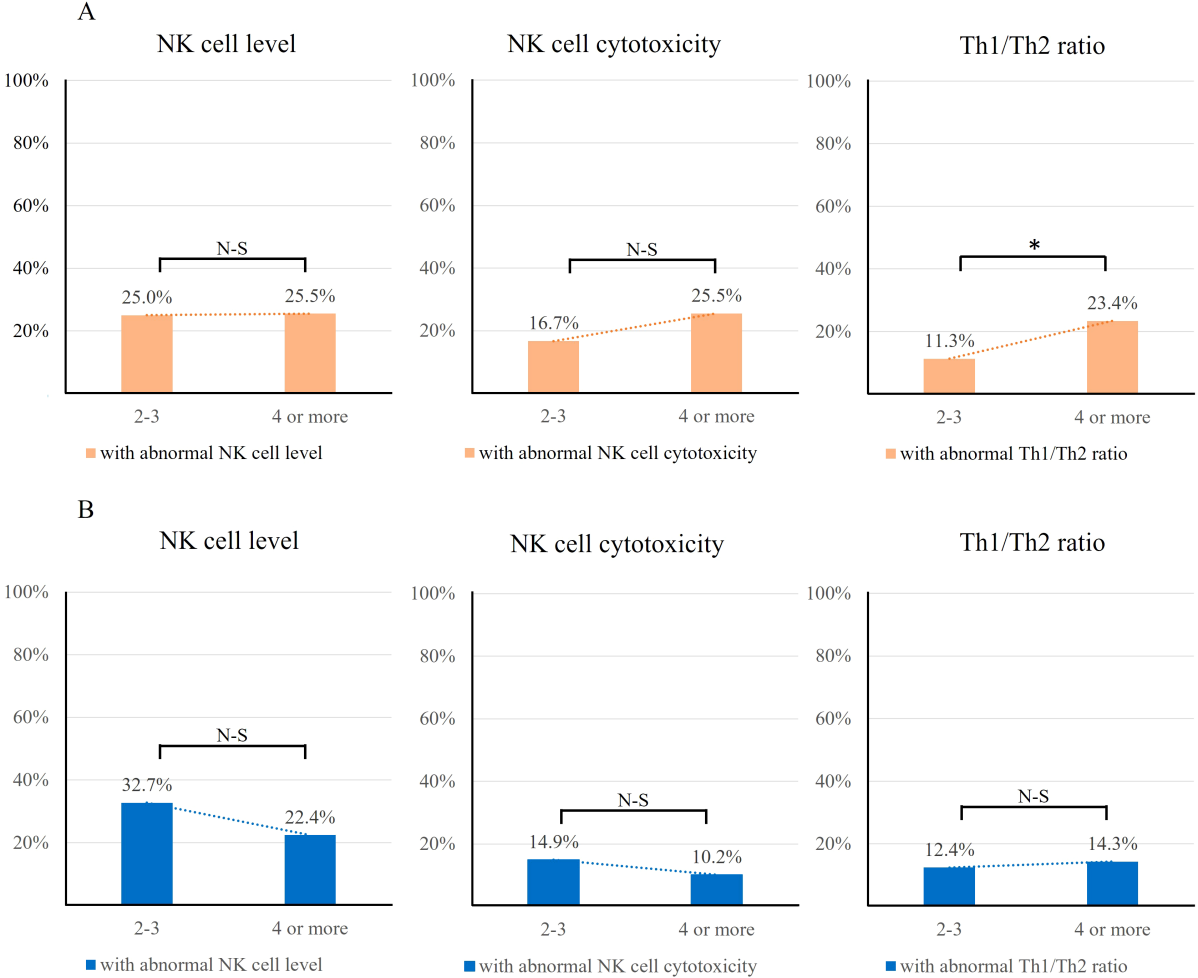
Correlation between each type of cellular immunity and number of previous miscarriages. **(A)** known etiology group. The prevalence of an elevated Th1/Th2 ratio was significantly higher in the group with four or more miscarriages. However, neither NK cell levels nor NK cell cytotoxicity showed a significant correlation with the number of miscarriages. (*P<0.05). **(B)** Idiopathic group. None of the abnormal cellular immunity showed correlation with the number of miscarriages. N-S, Not significant.

We explored the relationship between different types of cellular and the number of previous miscarriages in known and idiopathic groups. Intriguingly, a significant correlation between the number of miscarriages and a specific type of cellular immunity was found in the known etiology group, where abnormal Th1/Th2 ratio prevalence increased with the number of miscarriages (11.3% versus 23.4%, P=0.034) (**Figure 6A**). However, any kinds of trends were not observed in idiopathic group (**Figure 6B**).

### 3.8 Fetal aneuploidy rate according to number of miscarriages

**Figure 7:**
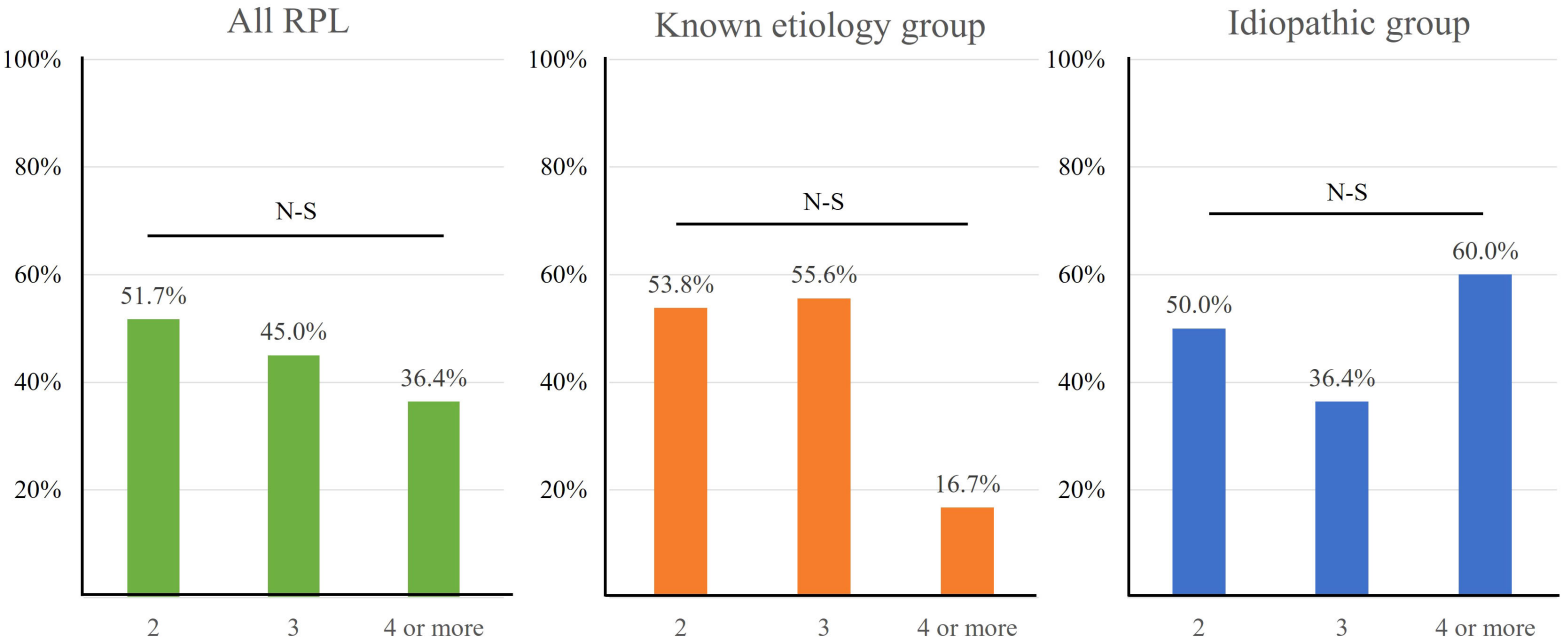
Fetal aneuploidy rate according to previous number of miscarriages. When miscarriage was confirmed in the index pregnancy, a fetal chromosomal test was performed with patient consent. The results were analyzed across three groups. In the all RPL group, there was a trend suggesting that the aneuploidy rate decreased as the number of previous miscarriages increased, although this trend was not statistically significant. Neither the known etiology group nor the idiopathic group showed any correlation between the number of miscarriages and the aneuploidy rate. N-S, Not significant.

Although not all patients who had miscarriages in index pregnancy underwent fetal chromosome testing (60 tests in 90 miscarriages), an analysis into aneuploidy rates showed a decreasing pattern in the all RPL group, though not statistically significant. (P=0.674) (**Figure 7**) (**Table 3**) Similarly, there were no significant associations between fetal aneuploidy and number of previous miscarriages in known etiology and idiopathic groups.

**Table 3:** Euploidy miscarriage rate and treatment failure.

	Cellular Immunity	Miscarriages (n)	Fetal Chromosome Test (n)	Fetal Euploidy (%)	P-value	Treatment Failure (%)	P-value
Known etiology group	Normal (n=118)	22	14	7 (50.0%)	0.382	7/110 (6.4%)	0.673
	Abnormal (n=97)	16	14	8 (57.1%)		6/95 (6.3%)	
Idiopathic group	Normal (n=151)	30	21	10 (47.6%)		11/142 (7.7%)	
	Abnormal (n=100)	22	11	7 (63.6%)		4/89 (4.5%)	

RPL, Recurrent pregnancy loss, Treatment failure rate =number of euploidy abortions/(Total n of pregnancies-fetal chromosome test not done)*100.

### 3.9 Euploidy miscarriage rate and treatment failure

The euploidy miscarriage rate and treatment failure rate were detailed in **Table 3**. Despite limitations in the study design and incomplete fetal chromosome testing data, the treatment failure rate, calculated using the equation as reported before: treatment failure rate=number of euploidy abortions/(total n of pregnancies-fetal chromosome test not done)*100 (32). Treatment failure rate ranged from 4.5% to 7.7%. While the treatment failure rate appeared higher in the idiopathic group, no significant differences were noted.

## 4 Discussion

RPL has multifactorial causes, and this study focused on immune imbalance as a potential factor. To uncover the underlying cellular immune abnormalities contributing to RPL, we examined the role of three immune markers we developed: NK cell levels, NK cell cytotoxicity, the Th1/Th2 ratio in peripheral blood, and the efficacy of IVIG treatment based on the immune marker abnormality. Our findings suggest that IVIG therapy may improve LBRs, particularly in those with specific immune abnormalities.

Consistent with previous study (23), considerable cellular immune abnormalities were also identified among women with known etiologies (**Figure 2**). Therefore, if pregnancy loss persists despite appropriate management of known etiologies, it is advisable to assess cellular immune function to further improve pregnancy outcomes.

In this study, when IVIG was used to modulate the immune response in women with RPL with abnormal cellular immune immunity, the pregnancy outcomes were comparable to those in women without abnormal cellular immunity, with LBRs of 82.7% versus 80.7%, respectively (**Figure 3A**). This suggests that IVIG can improve adverse pregnancy outcomes caused by an excessively activated cellular immune response. This finding aligns with previous research by several teams, including our own clinic (29, 32, 40). Similarly, Ahmadi et al. found that IVIG treatment could modulate peripheral blood Th17 and regulatory T cells, potentially contributing to improved pregnancy outcomes.

An early meta-analysis investigating IVIG effectiveness in RPL did not show significant benefits for women with primary RPL, highlighting the need for further research to establish its efficacy (41). The major limitation of this study was that it included participants who received IVIG without prior immune testing. More recent studies have shown better outcomes, as physicians began using IVIG in patients diagnosed with immune abnormalities. Winger et al. demonstrated that IVIG can significantly improve pregnancy outcomes in reproductive failure (RF) women with elevated NK cell cytotoxicity and Th1/Th2 ratio who underwent *in vitro* fertilization, emphasizing the importance of immune testing in guiding treatment (33). Moreover, a systematic review and meta-analysis focusing on the role of NK cells in female infertility and RPL found significantly higher percentages and numbers of NK cells in women with RM compared to controls (43). Also, another systematic review and meta-analysis assessed the impact of immunotherapy, including IVIG, in reproductive failure women with abnormal NK cell levels or activity. One of their key findings was that IVIG reduced NK cell activity and improved LBRs in women with RPL, as well as clinical pregnancy rates in women with RIF. However, the study also reported substantial heterogeneity (I² = 62%), highlighting the limitations of the current evidence and the need for higher-quality trials (34). Additionally, a recent meta-analysis of IVIG in RPL by Shi et al. demonstrated the potential benefits of IVIG, particularly for women with RPL and immune abnormalities (35). On the other hand, a meta-analysis points out the weakness as there is a clinical heterogeneity of the included studies, which results in inconsistency in outcomes across different trials (43). Varying definitions of immune abnormalities might contribute to these discrepancies. This variability emphasizes the importance in standardized patient selection and treatment protocols better to define the role of IVIG in RPL management.

In our study, patients were stratified into subgroups based on three commonly used cellular immune markers: NK cell proportion, NK cell cytotoxicity, and Th1/Th2 cytokine ratio. These markers are frequently assessed in clinical practice and are routinely used at our institution to guide immunological evaluation and patient counseling regarding IVIG treatment. To our knowledge, this is the first study to comprehensively evaluate pregnancy outcomes in RPL patients by simultaneously considering all three immune parameters (**Figure 3B**). We believe this stratified approach offers a more refined understanding of how specific immune abnormalities may influence the therapeutic efficacy of IVIG. In our study, notably, there was no statistical difference in LBRs among all eight subgroups (21, 32, 46), suggesting that the type or combination of abnormal cellular immunity factors does not affect pregnancy outcomes. Our study adds to this body of evidence by providing a comprehensive analysis of multiple immune parameters, further supporting the hypothesis that targeted immune modulation may be beneficial. Pregnancy outcomes with IVIG treatment were not inferior to those patients with no cellular immune abnormalities. This suggests that IVIG can potentially mitigate the adverse impact of immune dysfunction on pregnancy outcomes. Nonetheless, these findings should be interpreted with caution, and further studies with larger sample sizes are warranted. Moreover, we used the widely adopted IVIG dosage of 0.4 g/kg, as commonly applied in reproductive immunology, which may appear low compared to the higher doses typically used in certain autoimmune or hematologic disorders(e.g., 1 g/kg) (21, 28, 30, 32, 45, 47). Although few groups proposed higher dose regimens to treat RPL women (2, 36), no studies to date have directly compared the efficacy of high-dose versus low-dose IVIG regimens. Further investigations are needed to establish the optimal dosing strategy.

Interestingly, an increase in age and BMI negatively impacted the LBRs, particularly in the idiopathic RPL group (**Table 2**). Even when underlying causes were addressed and abnormal cellular immunity was treated, negative pregnancy outcomes could still occur by aging and increasing BMI. Thus, for successful pregnancy outcomes, patients with obesity should be encouraged for weight reduction, and should be counseled not to delay pregnancy.

We examined the LBR in women with RPL as the number of miscarriages increased. Although no significant trend was observed, the graph indicates a general plateau, even among women with four or more miscarriages (all RPL: 78.1%, known etiology: 78.7%, idiopathic: 77.6%). Previously, it was widely understood that LBR declined as the number of miscarriages increased. A study reported a decline in LBR below 70% after three miscarriages (three miscarriages: 67.6%, four miscarriages: 63.0%, five miscarriages: 51.3%, six or more miscarriages: 25.2%) (48). In a recent RCT that included women with four or more miscarriages of unknown etiology, the ongoing pregnancy rate was significantly higher in the IVIG treatment group compared to the placebo group (58.0% vs. 34.7%, p=0.03), suggesting a beneficial effect of IVIG therapy (2). This finding may offer insights into our results, indicating that IVIG treatment could improve pregnancy outcomes in women with a higher number of miscarriages. Thus, we propose that thorough evaluation of immune abnormalities and appropriate treatment have contributed to improving pregnancy outcomes.

We hypothesized that the prevalence of abnormal cellular immunity would increase with the number of previous miscarriages (**Figure 5**). Notably, both the all RPL group and the known etiology group demonstrated a significant increase in abnormal cellular immunity as the number of prior miscarriages increased. In contrast, no such trend or statistical significance was observed in the idiopathic group. This increasing trend in abnormal cellular immunity with the number of miscarriages underscores the importance of conducting a cellular immunity workup in the known etiology group. Even with appropriate treatment for the identified cause of RPL, patients may still have underlying cellular immunity issues that require further attention. However, this point warrants further investigation, ideally with a larger sample size. Moreover, we investigated the correlation between each type of cellular immunity and the previous number of miscarriages. In the known etiology group, the prevalence of elevated Th1/Th2 ratio was significantly high in four or more miscarriages group (**Figure 6A**, p=0.034). These results are in line with the updated ESHRE guideline 2023 which recommends IVIG treatment strictly for women with four or more unexplained RPL (49). Additionally, as our previous study reported an increase in the Th1/Th2 ratio with advancing age, we aimed to verify whether the prevalence of an elevated Th1/Th2 ratio correlated with the number of prior miscarriages (50). However, the mean age was not different between the two groups (2–3 miscarriages group:32.4, 4 or more miscarriages group:33.2, p=0.525) Thus, the increased prevalence of an elevated Th1/Th2 ratio may indicate the intrinsic pathophysiology of recurrent pregnancy loss.

Previous reports have demonstrated an increased prevalence of abnormal fetal karyotypes with fewer previous miscarriages. Ogasawara et al. showed that as the number of previous miscarriages increased, the percentage of chromosomal anomalies decreased, indicating an underlying cause of repeated miscarriages (51). Lee et al. also found that women with 2–3 abortions had a higher prevalence of aneuploidy than those with four or more previous abortions (32). In this study, we also sought to find this trend, but the results were inconsistent. There may have been a selection bias as we collected data from only those with pregnancy outcomes. Also, not all patients who experienced miscarriages took karyotype evaluation of the abortus, resulting in a limited number of cases with fetal karyotype data. Nevertheless, fetal karyotyping is crucial for identifying the cause of miscarriage in RPL patients, as confirming aneuploidy helps plan and prepare for subsequent pregnancies. After excluding patients with abnormal fetal karyotypes, clinicians should focus on addressing maternal causes.

A key strength of this study is its comprehensive evaluation of a large cohort of women with RPL over an extended period. By including only those patients with a complete diagnostic workup and known treatment outcomes, the study minimizes bias related to incomplete data, enhancing the reliability of the findings. Additionally, the study’s detailed stratification of patients based on specific immune markers (NK cell proportion, NK cell cytotoxicity, and Th1/Th2 ratio) allows for a more nuanced understanding of how different immune abnormalities may impact pregnancy outcomes. This granularity is valuable for developing more targeted treatment strategies. Furthermore, the use of well-established cutoff values for defining abnormal cellular immunity, derived from previous research conducted at our clinic, ensures consistency and comparability in identifying patients at risk for immune-mediated pregnancy loss. Moreover, to reduce bias, only the first pregnancy outcomes after the baseline evaluation were included in this study.

This study has several limitations. First, it was designed retrospectively, which inherently introduces challenges, including the potential for numerous follow-up losses and incomplete data collection. Second, the study was conducted at a single center, which may limit the generalizability of our findings to other populations or healthcare settings. The single-center design also raises concerns about the potential for site-specific biases that could influence the outcomes observed. Moreover, it should be noted that in the group with known etiologies, pharmacologic interventions such as corticosteroid, LDA, LMWH, and levothyroxine were administered as part of clinical management. Some of these agents are known to exert immunomodulatory effects, which may have contributed to the observed outcomes. Although corticosteroids were rarely used in this study, the potential influence of these co-administered treatments cannot be fully excluded and should be taken into account when interpreting the effects of IVIG in this subgroup. Lastly, the stratification into multiple immune subgroups may introduce bias and reduce statistical power. However, this reflects clinical heterogeneity and enables a more precise understanding of how different immune abnormalities may influence treatment outcomes.

On conclusion, IVIG treatment can be a potential therapeutic agent to improve LBRs in women with RPL and abnormal cellular immunity. However, prospective studies are warranted to elucidate the efficacy of IVIG treatment, refine patient selection criteria, and optimize treatment protocols to improve pregnancy outcomes in this population.

## Data Availability

The raw data supporting the conclusions of this article will be made available by the authors, without undue reservation.
